# Genotypic and Phenotypic Resistance Profiles of Invasive *Acinetobacter baumannii* Isolates at Bulgarian University Hospital

**DOI:** 10.3390/microorganisms14081618

**Published:** 2026-07-24

**Authors:** Teodora Marinova-Bulgaranova, Preslava Hristova, Polya Marinovska, Nikolay Balgaranov, Vladislav Nankov, Slavil Peykov, Hristina Hitkova

**Affiliations:** 1 Department of Microbiology and Virology, Medical University of Pleven, 5800 Pleven, Bulgaria; preslava.hristova@mu-pleven.bg (P.H.); polya.marinovska@mu-pleven.bg (P.M.); 2 Department of Pediatric Diseases, Medical University of Pleven, 5800 Pleven, Bulgaria; nikolai.balgaranov@mu-pleven.bg; 3 Department of Anatomy, Histology, Cytology and Biology, Medical University Pleven, 5800 Pleven, Bulgaria; vladislav.nankov@mu-pleven.bg; 4 Department of Medical Microbiology, Faculty of Medicine, Medical University of Sofia, 2 Zdrave Str., 1431 Sofia, Bulgaria; spejkov@biofac.uni-sofia.bg; 5 Department of Genetics, Faculty of Biology, University of Sofia ‘St. Kliment Ohridski’, 8 Dragan Tzankov Blvd., 1164 Sofia, Bulgaria; 6 BioInfoTech Laboratory, Sofia Tech Park, 111 Tsarigradsko Shose Blvd., 1784 Sofia, Bulgaria

**Keywords:** *A. baumannii*, minimal inhibitory concentration, antimicrobial resistance genes, resistance genotype, resistance phenotype

## Abstract

*Acinetobacter baumannii* is a formidable nosocomial pathogen responsible for severe hospital-acquired infections. This study aimed to investigate the relationship between genotypic and phenotypic resistance profiles in 36 non-duplicate invasive *A. baumannii* isolates collected at a tertiary care hospital in Bulgaria over a six-year period (2019–2024). Identification was performed by MALDI-TOF MS and PCR-based detection of *bla*_OXA-51-like_ and *gyrB*. Antimicrobial susceptibility testing to ten agents was performed by gradient diffusion and broth microdilution, and selected resistance genes were detected by conventional PCR. Whole-genome sequencing of 17 representative isolates was conducted to confirm the resistance genotype–phenotype associations and perform MLST. Carbapenem resistance was detected in 34/36 isolates (94.4%), mediated by *bla*_OXA-23-like_ (29/34) or *bla*_OXA-72_ (5/34). The *armA* gene associated with high-level resistance to all three aminoglycosides was identified exclusively in *bla*_OXA-23-like_-positive isolates (18/29). In *armA*-negative isolates, gentamicin and tobramycin MICs decreased markedly, whereas amikacin MICs remained elevated. In addition, minocycline MICs ≥ 8 mg/L were observed in the presence of *armA*. Seven distinct genotypic–phenotypic profiles were defined based on these gene–MIC associations. Resistome and phylogenetic analyses confirmed these relationships, with MLST assigning the isolates to established clonal lineages ST2 and ST636 (International Clone II). These findings support the use of targeted genes, such as *armA*, combined with phenotypic testing to guide empirical therapy and strengthen local surveillance of high-risk *A. baumannii* clones in hospital settings.

## 1. Introduction

*Acinetobacter baumannii* has emerged over the past decade as a major nosocomial pathogen owing to its capacity to withstand last-line antimicrobials and disseminate clonally [1,2]. As a member of the ESKAPE group (*Enterococcus faecium*, *Staphylococcus aureus*, *Klebsiella pneumoniae*, *A. baumannii*, *Pseudomonas aeruginosa*, and *Enterobacter* spp.), it ranks among the most critical threats to global public health [3]. Its resistance to desiccation enables prolonged survival in the hospital environment, thereby facilitating endemic persistence [2].

The European Antimicrobial Resistance Surveillance Network (EARS-Net) data for 2024 indicate that carbapenem resistance in *A. baumannii* exceeds 90% in several Southeast European EU Member States, particularly in Balkan countries [4]. Moreover, the Balkan region showed the highest rates of combined resistance: Romania (89.5%), Croatia (89.0%), and Greece (87.9%) [4]. In Bulgaria, the proportion of invasive carbapenem-resistant *A. baumannii* (CRAB) isolates increased from 80% in 2022 to 90.5% in 2024, following the COVID-19 pandemic period [4]. During the same period, combined resistance rose from 75.0% to 86.7%, markedly exceeding the EU/EEA population-weighted mean of 30.2% in 2024 [4].

Antibiotic resistance in Gram-negative bacteria is mediated by three principal strategies: enzymatic inactivation or complete hydrolysis of the antimicrobial molecule, modification of the target, and reduced membrane permeability resulting from the loss or alteration of porins and/or the activity of efflux pumps [5,6]. These mechanisms operate in clinical *A. baumannii* isolates and are driven by well-defined genetic determinants or mutations. In this regard, it is important to highlight the remarkable genomic plasticity of this species, which facilitates chromosomal rearrangements and the integration of foreign genetic elements [7]. This intrinsic adaptability, coupled with antibiotic selective pressure, has contributed to the establishment of endemic multidrug-resistant *A. baumannii* strains in healthcare settings [8].

Since the introduction of sequencing technologies in microbiology, considerable efforts have been devoted to elucidating the mechanisms underlying multidrug resistance in *A. baumannii*. The genomes of both collection and endemic strains have been subjected to detailed comparative analysis [9,10,11]. Resistance islands, plasmids, and mobile genetic elements have been identified and characterized [9,10,11,12], while the genetic environment of resistance determinants has been studied in detail [11,13]. Based on these investigations, the authors have drawn conclusions regarding the dissemination of genetic determinants among strains and species.

Fournier et al. [9] reported the presence of genes within a resistance island acquired from *Pseudomonas*, *Salmonella*, and *Escherichia* species.

Zhu et al. [11] concluded that horizontal gene transfer of mobile genetic elements between chromosomes and plasmids may be a significant contributor to the rapid spread of resistance among *A. baumannii* strains.

Holt et al. [14] provided experimental evidence that natural transformation is a major driver of resistance gene acquisition in Global Clone 1.

Frenk et al. [8] observed that sequence type and clade distribution remained stable over time, suggesting that horizontal gene transmission and the de novo emergence of multidrug-resistant *A. baumannii* are rare events, yet crucial to the evolutionary success of specific clades and sequence types.

To date, large-scale systematic investigations of invasive *A. baumannii* isolates integrating phenotypic and genotypic data remain scarce in Bulgaria. The present study aimed to characterize the prevalent phenotypes and genotypes among 36 invasive *A. baumannii* isolates recovered from inpatients at a university hospital over a six-year period (2019–2024) and elucidate the relationships between phenotypic resistance profiles and the underlying genetic determinants.

## 2. Materials and Methods

### 2.1. Bacterial Isolates

During the study period (2019–2024), a total of 67 non-duplicate *Acinetobacter calcoaceticus–baumannii* complex (Acb) isolates were recovered from blood cultures at the University Hospital. The isolates were routinely identified to the Acb-complex level using the VITEK 2 Compact system (bioMérieux, Marcy-l’Étoile, France) and tested for antimicrobial susceptibility by disk diffusion and VITEK 2. They were then stored in skim-milk broth at −80 °C pending further study. From these 67 non-duplicate isolates, 36 (53.7%) were selected for further investigation. The selection was based on two criteria: (i) representation of every calendar year of the study period and (ii) coverage of defined susceptibility phenotypes, based on the routine susceptibility results for eight agents (MEM, IMI, GEN, AMK, TOB, CIP, LEV, and COL) (Supplementary Table S1). All rare phenotypes were retained, whereas the predominant phenotypes were proportionally subsampled to reduce their overrepresentation relative to the rarer phenotypes, resulting in the exclusion of 31 isolates from further analysis. One of the two isolates susceptible only to tobramycin and colistin was additionally excluded owing to non-viability, yielding a final collection of 36 isolates. For the cohort of 36 selected isolates, species-level identification was confirmed by MALDI-TOF MS (VITEK MS, bioMérieux, Marcy-l’Étoile, France) and two PCR-based methods targeting the *bla*_OXA-51-like_ gene [15] and *gyrB* [16]. *A. baumannii* ATCC 19606 was used as the positive control strain for PCR-based species identification.

The study was approved by the Ethics Committee of the Medical University of Pleven (Approval No. 794, approved in June 2024) and conducted in accordance with the Declaration of Helsinki and its later amendments. As the research focused solely on bacterial isolates and no personal patient information was used, individual informed consent was not required.

### 2.2. Antimicrobial Susceptibility Testing

Minimum inhibitory concentrations (MICs) were determined by the gradient diffusion method (MIC Test Strip, Liofilchem, Roseto degli Abruzzi, Italy) for imipenem (IPM), meropenem (MEM), gentamicin (GEN), amikacin (AMK), tobramycin (TOB), levofloxacin (LEV), trimethoprim/sulfamethoxazole (SXT), and minocycline (MIN). MICs for cefiderocol (FDC) and colistin (COL) were determined by the broth microdilution method using ComASP™ Cefiderocol (0.008–128 mg/L) and ComASP™ Colistin (0.25–16 mg/L) panels (Liofilchem, Roseto degli Abruzzi, Italy), respectively. *Escherichia coli* ATCC 25922, *P. aeruginosa* ATCC 27853, and *A. baumannii* ATCC 19606 served as quality control strains. MICs were interpreted according to EUCAST clinical breakpoints (v16.0, 2026). For minocycline, CLSI criteria( M100, 35th edition, 2025) [17] were applied.

### 2.3. Definition for MDR-AB, XDR-AB, and PDR-AB

Multidrug-resistant (MDR) *A. baumannii* isolates were defined as non-susceptible to at least one agent in three or more antimicrobial categories, whereas extensively drug-resistant (XDR) isolates were defined as non-susceptible to at least one agent in all but two or fewer antimicrobial categories. Pandrug-resistant (PDR) isolates were defined as non-susceptible to all agents in all antimicrobial categories, according to previously described criteria [18].

### 2.4. DNA Isolation

Total DNA from all investigated strains was isolated using the DNeasy Blood and Tissue Kit (QIAGEN, Hilden, Germany), according to the manufacturer’s instructions, from 3 mL of overnight culture inoculated with a single colony.

### 2.5. PCR Screening for Antimicrobial Resistance Genes (ARGs)

Amplifications were performed on a qTOWER^3^ G touch thermal cycler (Analytik Jena, Jena, Germany). Genes encoding carbapenemase production (*bla*_OXA-23-like_, *bla*_OXA-24/40-like_, *bla*_OXA-58-like_, *bla*_NDM-like_, *bla*_VIM-like_, *bla*_IMP-like_, and *bla*_KPC-like_) were investigated. The specific allelic variant within the *bla*_OXA-24/40-like_ group was subsequently determined by whole-genome sequencing (WGS) (see Section 3.7, Resistome Analysis). The association of IS*Aba1* with *bla*_OXA-23-like_ was assessed by PCR using an IS*Aba1*-specific forward primer and a *bla*_OXA-23-like_-specific reverse primer. In addition, PCR assays were performed for the *armA* gene, encoding a 16S rRNA methylase associated with high-level aminoglycoside resistance, and the *sul1* gene, associated with sulfonamide resistance and located within class 1 integrons.

PCR assays were performed using previously described protocols with minor modifications, mainly to the annealing temperatures [18,19,20,21,22,23]. All reactions were carried out in a final volume of 20 µL containing OneTaq^®^ 2× Master Mix (New England Biolabs, Ipswich, MA, USA) and 20 ng of template DNA. The class D carbapenemase genes were detected in a multiplex hot-start reaction (94 °C for 4 min; 36 cycles of 94 °C for 30 s, 52 °C for 35 s, 70 °C for 75 s; final extension 71 °C for 3 min). The *bla*_VIM-_, *bla*_IMP-_, *bla*_NDM-_, and *bla*_KPC-like_ genes were amplified by touchdown PCR (95 °C for 3 min; 5 cycles with the annealing temperature decreasing by 1.6 °C per cycle from 63 °C, followed by 20–25 cycles at 54 °C; extension 70 °C for 1 min). The *armA* gene was amplified at an annealing temperature of 50 °C (30 cycles), and *sul1* by touchdown PCR (62 °C decreasing by 1 °C per cycle for 7 cycles, followed by 25 cycles at 54 °C). The IS*Aba1*–*bla*_OXA-23-like_ assay was performed at 52 °C for 30 cycles. The expected amplicon size of the IS*Aba1*–*bla*_OXA-23-like_ PCR assay was verified in silico using Primer-BLAST (National Center for Biotechnology Information (NCBI), Bethesda, MD, USA; https://www.ncbi.nlm.nih.gov/tools/primer-blast/, accessed on 4 July 2025).

Primer sequences and expected amplicon sizes are provided in Table 1. PCR products were separated by agarose gel electrophoresis and visualized by ethidium bromide staining. The following strains were used as controls:

- *P. aeruginosa* 4782 (*bla*_IMP-100_/bla_VIM-4_)
- *P. aeruginosa* 1793 (*bla*_NDM_/bla_GES_)
- *K. pneumoniae* ATCC 2814 (*bla*_KPC_)
- *A. baumannii* strains (*bla*_OXA23-like_/*bla*_OXA24/40-like_)
- *A. baumannii* (*bla*_OXA58_)

The *A. baumannii* strains are in-house reference strains provided by the National Center of Infectious and Parasitic Diseases (NCIPD), Sofia, Bulgaria, previously characterized by PCR and sequencing.

### 2.6. Whole-Genome Sequencing (WGS)

Based on the combined phenotypic susceptibility and PCR screening results, seven distinct genotypic–phenotypic resistance profiles were defined. A subset of 17 isolates, representative of these profiles, was selected for WGS to enable comprehensive resistome analysis. Genomic DNA was randomly fragmented, size-selected, ligated to adapters, and PCR-amplified to generate sequencing libraries. The libraries were sequenced on an Illumina NovaSeq X Plus platform (Novogene, Cambridge, UK), producing 2 × 150 bp paired-end reads.

### 2.7. Draft Genome Assembly

The genome assembly procedure involved several steps: quality control (FastQC v0.12.1, https://www.bioinformatics.babraham.ac.uk/projects/fastqc/, accessed on 15 April 2026), raw read preprocessing (Trimmomatic v0.39) [25], and genome assembly (SPAdes v4.2.0; --isolate and --cov-cutoff 5.0 flags) [26]. The resulting scaffold sequences were uploaded to the Galaxy online platform [27], where draft genome metrics were evaluated (Galaxy v5.3.0). All software tools were run with default parameters unless otherwise specified.

### 2.8. Resistome and Multilocus Sequence Typing (MLST) Analyses

All uploaded draft genome sequences were screened for AMR genes using the ABRicate tool (Galaxy v1.4.0) with the following settings: the Comprehensive Antibiotic Resistance Database (CARD), minimum DNA identity of 70%, and minimum DNA coverage of 60%. In addition, the *gyrA* and *parC* coding sequences were manually analyzed for mutations in their quinolone resistance-determining regions (QRDRs).

Finally, MLST analysis was performed on the assembled scaffolds using the MLST tool (Galaxy v2.22.0, https://usegalaxy.eu/) and the two available *A. baumannii* schemes.

### 2.9. Phylogenomic Analysis

Clinical *A. baumannii* genomes were retrieved from the Isolates Browser integrated into the NCBI Pathogen Detection system for Albania, Bosnia and Herzegovina, Bulgaria, Croatia, Greece, Kosovo, Montenegro, North Macedonia, Romania, Serbia, and Turkey. To minimize sampling bias, a single representative genome was selected from each available single-nucleotide polymorphism (SNP) cluster for every country, preferentially choosing bloodstream isolates when available. All selected draft genome sequences were annotated using Prokka v1.14.6 [28].

Pangenome analysis was subsequently performed using Roary v3.13.0 [29] with a minimum BLASTP identity threshold of 95% and a core genome definition of 99%, generating a core gene alignment for downstream analyses. All SNPs were extracted from the resulting multi-FASTA alignment using the Find SNP Sites tool (Galaxy v2.5.1), and pairwise SNP distances were calculated using the SNP Distance Matrix tool (Galaxy v0.8.2). The resulting distance matrix was subjected to hierarchical clustering using the Python data visualization library seaborn (v0.13.2).

A phylogenetic tree was reconstructed from the core genome SNP alignment using RAxML v8.2.12 [30], with branch support estimated from 1000 bootstrap replicates. The resulting tree was visualized using the Interactive Tree of Life (iTOL) web server [31].

### 2.10. Statistical Analysis

Statistical analyses were conducted using jamovi (v2.7; The jamovi Project, 2025) [32], based on R (v4.5; R Core Team, 2025) [33]. Associations between the presence of resistance genes and antimicrobial susceptibility were assessed using Fisher’s exact test for categorical variables. Differences in MIC values between gene-positive and gene-negative groups were assessed using the Mann–Whitney U test. Differences among aminoglycosides within *armA*-negative, *bla*_OXA-23-like_-positive isolates were assessed using the Friedman test, with pairwise comparisons by Wilcoxon signed-rank test. A *p*-value < 0.05 was considered statistically significant. Genotype subgroups with very small sample sizes (n ≤ 4) were summarized descriptively, without formal statistical testing, owing to insufficient statistical power.

## 3. Results

### 3.1. Patient Demographic and Clinical Characteristics

Supplementary Table S2 presents the demographic and clinical characteristics of the 36 patients (16 men, 20 women). The age distribution by category was as follows: 0–18 years (1/36), 19–40 years (6/36), 41–65 years (17/36), and >65 years (12/36). Most patients (28/36) were treated in intensive care units, followed by surgical units (6/36), with one case each in the Gastroenterology Clinic and the Acute Cardiac Care Unit. The mean length of hospital stay was 22.6 days. Patients were diagnosed with polytrauma, including one case of second-degree burns and one of toxic gas exposure (8/36); acute abdominal disease, including peritonitis, pancreatitis, ileus, colon malignancies with complications, and liver failure (11/36); vascular and cardiac disease (5/36); pulmonary infections (6/36), five of them associated with COVID-19; soft-tissue infections (3/36); systemic lupus erythematosus (1/36); thrombocytopenia (1/36); and viral meningitis (1/36). Prior to the isolation of *A. baumannii*, broad-spectrum antibiotic therapy was documented in 32/36 patients. Mortality was high (26/36), with 10 patients discharged following clinical improvement.

### 3.2. Identification

A total of 36 invasive isolates were confirmed as *A. baumannii* by MALDI-TOF MS (VITEK MS) and PCR-based methods targeting the intrinsic *bla*_OXA-51_-like gene and the *gyrB* gene.

### 3.3. Antimicrobial Susceptibility

The results of antimicrobial susceptibility testing are presented in Table 2. Carbapenem resistance was observed in 34 of 36 isolates (94.4%), with MICs of ≥32 mg/L for both meropenem and imipenem. All isolates were susceptible to colistin. With respect to aminoglycosides, resistance rates varied by agent. Gentamicin showed the lowest resistance, with 16/36 isolates (44.4%) categorized as susceptible and 20/36 (55.6%) as resistant. In contrast, tobramycin and amikacin resistance was considerably higher, with only 6/36 (16.7%) and 2/36 (5.6%) isolates susceptible and 30/36 (83.3%) and 34/36 (94.4%) resistant, respectively. Resistance to trimethoprim/sulfamethoxazole was observed in 35/36 isolates (97.2%). Non-susceptibility (resistant plus intermediate) to levofloxacin was observed in 35/36 isolates (97.2%). Minocycline resistance (MIC range 8–24 mg/L) was observed in 18/36 isolates (50.0%), 10/36 (27.8%) showed intermediate susceptibility (MIC range 1.5–2 mg/L), and 8/36 (22.2%) were susceptible (MIC ≤ 1 mg/L).

As an exploratory sub-analysis, cefiderocol susceptibility testing was performed on a subset of nine isolates, selected to represent different genotypic profiles identified in this study. MIC values were interpreted using the non-species-specific PK/PD cutoff (S ≤ 2 mg/L, EUCAST v16.0). Seven of the nine isolates fell within this range, whereas two (MIC 4 mg/L) exceeded the PK/PD susceptibility cutoff.

Overall, 34/36 isolates (94.4%) met the criteria *for XDR* classification, remaining susceptible only to colistin. The findings regarding cefiderocol should be considered preliminary, given the limited number of isolates tested.

### 3.4. PCR Screening for Antimicrobial Resistance Genes

The intrinsic *bla*_OXA51-like_ determinant was detected in all 36 isolates (100%), confirming their identification as *A. baumannii*. The prevalence of *bla*_OXA23-like_ and *bla*_OXA24/40-like_ was 80.6% (29/36) and 13.9% (5/36), respectively. Using a primer combination consisting of the forward primer targeting IsaAba1 and reverse primer specific for *bla*_OXA23-like_, all *bla*_OXA23-like_ positive isolates yielded a 1404 bp amplicon, indicating the presence of IS*Aba1* upstream of the gene and suggesting promoter-mediated activation. No other *bla* genes were detected. The *armA* gene, encoding a 16S rRNA methylase, was identified in 50.0% (18/36) of the isolates. Notably, it was detected exclusively in combination with *bla*_OXA23-like_. No isolate simultaneously harbored *bla*_OXA24/40-like_ and *armA*, and no isolate harbored *armA* as a sole resistance determinant. The *sul1* gene, associated with resistance to sulfamethoxazole, was detected in 80.6% (29/36) of the isolates.

### 3.5. Association Between Resistance Phenotypes and Genotypes

Table 3 summarizes the relationship between antimicrobial resistance phenotypes and the detected resistance genes. The complete MIC values and detected resistance genes for all 36 isolates are provided in Table S2, and the detailed statistical output is in Table S3 (Supplementary Materials). The presence of either *bla*_OXA-23-like_ or *bla*_OXA-24/40-like_ was associated with high MIC values for meropenem and imipenem (≥32 mg/L), whereas their absence corresponded to MIC values within the susceptible range (Fisher’s exact test, *p* = 0.002).

The *armA* gene was associated with significantly higher MICs for all three aminoglycosides tested (Mann–Whitney U test, *p* < 0.001), with *armA*-positive isolates reaching 768–1024 mg/L for gentamicin, 256 mg/L for amikacin, and 512–1024 mg/L for tobramycin. In *armA*-negative, *bla*_OXA-23-like_-positive isolates, gentamicin MICs decreased to the susceptible or low-level resistance range (2–6 mg/L), and tobramycin MICs dropped markedly from ≥1024 to 12–24 mg/L (Mann–Whitney U test, *p* < 0.0001 for both). Amikacin MIC values, however, remained elevated compared with gentamicin and tobramycin (Friedman test, *p* < 0.0001; pairwise Wilcoxon signed-rank tests, *p* = 0.001 for all comparisons).

Notably, elevated minocycline MICs were also significantly associated with *armA* carriage (Mann–Whitney U test, *p* < 0.001; 8–24 mg/L), and a strong concordance was observed between *armA* carriage and minocycline resistance (Fisher’s exact test, *p* < 0.001), with susceptibility to minocycline observed only among *armA*-negative isolates. These findings suggest a potential co-linkage between *armA* and minocycline resistance determinants. Similar associations extended to fluoroquinolones: among the 29 *bla*_OXA-23-like_-positive isolates, those additionally carrying *armA* (18/29) showed significantly higher levofloxacin MICs (median 16, range 1–32 mg/L) than *armA*-negative *bla*_OXA-23-like_ carriers (11/29; median 1, range 1–8 mg/L; Mann–Whitney U test, *p* < 0.001), with a corresponding significant association between *armA* carriage and levofloxacin resistance (Fisher’s exact test, *p* = 0.001).

A significant difference was also found between *armA*-negative isolates harboring *bla*_OXA-23-like_ (11/29) and *bla*_OXA-24/40-like_-positive ones (5/36, all *armA*-negative). The former group showed significantly higher gentamicin MICs (median 4 vs. 2 mg/L; *p* = 0.003) and significantly higher tobramycin MICs (median 16 vs. 0.75 mg/L; *p* = 0.001). Amikacin MICs did not differ significantly between the two genotypes (*p* = 1.000), remaining elevated in both. Minocycline MICs were also significantly higher among *bla*_OXA-23-like_ carriers than among *bla*_OXA-24/40-like_-positive isolates (median 1.5 vs. 0.5 mg/L; *p* = 0.002).

The isolate carrying only *sul1* remained susceptible to carbapenems and amikacin and showed intermediate minocycline susceptibility and low-level resistance to gentamicin, tobramycin, and levofloxacin, but was highly resistant to trimethoprim/sulfamethoxazole (MIC ≥ 32 mg/L). The isolate lacking all screened genes was fully susceptible to all agents.

Among the nine isolates tested for cefiderocol, the *armA*-positive genotypes GT1 and GT3 showed MICs of 0.25–0.50 mg/L, whereas the *armA*-negative genotypes GT2, GT4, and GT6 showed higher values (1–4 mg/L). The gene-negative isolate (GT7) had the lowest MIC of the subset (0.125 mg/L). Given the limited size of the subset, this observation should be regarded as preliminary rather than a firm conclusion. In addition, these results were excluded from the general XDR susceptibility statement. Detailed cefiderocol MIC values by isolate and genotype are presented separately in Table S4 (Supplementary Materials).

### 3.6. Draft Genome Assembly and Evaluation

In total, 17 of the 36 isolates were subjected to whole-genome sequencing. The resulting data were used to perform MLST and resistome analysis. The draft genome assembly metrics are summarized in Table 4.

The genome sizes ranged from 3.93 to 4.42 Mbp, with a GC content of 39.01–41.25%. The N50 values ranged from 288,607 to 2,183,114 bp, and the number of contigs (≥1 kbp) varied from 15 to 97. Notably, isolate 120—the only susceptible isolate in the collection—exhibited markedly superior assembly metrics (only 15 contigs; N50 = 2,183,114 bp). This isolate could not be assigned to a sequence type by the Pasteur MLST scheme and was classified only by the Oxford MLST scheme as ST 1061.

### 3.7. Resistome Analysis

Table 5 presents the resistance determinants identified in the seven genotypes. *bla*_OXA-72_ was the only *bla*_OXA-24/40-like_ variant detected. All *armA*-positive isolates also harbored *tet(B)*, the main determinant for minocycline resistance. The *bla*_OXA-23-like_-positive isolates and the isolate positive only for *sul1* and lacking *armA* carried *aac(6′)-Ib7*, *aph(3′)-VIa*. One of these additionally harbored *aph(3′)-Ia*. The *bla*_OXA-24/40-like_-positive isolates carried only *aph(3′)-VIa*.

Comparison of the QRDRs of the isolates with the corresponding region of *A. baumannii* ATCC 19606 revealed amino-acid substitutions previously reported to play a role in quinolone resistance [34]. All but one isolate possessed S81L substitution in *GyrA*. The single isolate lacking this mutation was also the only one negative for all resistance genes screened by PCR. Isolates co-harboring *bla*_OXA-23-like_ and *armA*, as well as those positive for *bla*_OXA-72_, additionally carried S84L substitution in ParC.

### 3.8. MLST Analysis

MLST analysis (Pasteur scheme) assigned all but one of the sequenced isolates to either ST2 or its triple-locus variant ST636. Thirteen isolates were ST2, the globally dominant high-risk clone corresponding to International Clone II (IC2) [35,36,37], whereas three isolates, all harboring *bla*_OXA-72_ (a *bla*_OXA-24/40-like_ variant), were ST636. The remaining isolate could not be classified by the Pasteur scheme but was assigned to ST1061 by the Oxford scheme. It was negative for all tested acquired resistance genes, susceptible to all antimicrobials tested, and harbored two distinct intrinsic β-lactamase genes: blaADC-7 and blaOXA-314.

### 3.9. Phylogenomic Analysis of Clinical A. baumannii Isolates from the Balkans

**Figure 1:**
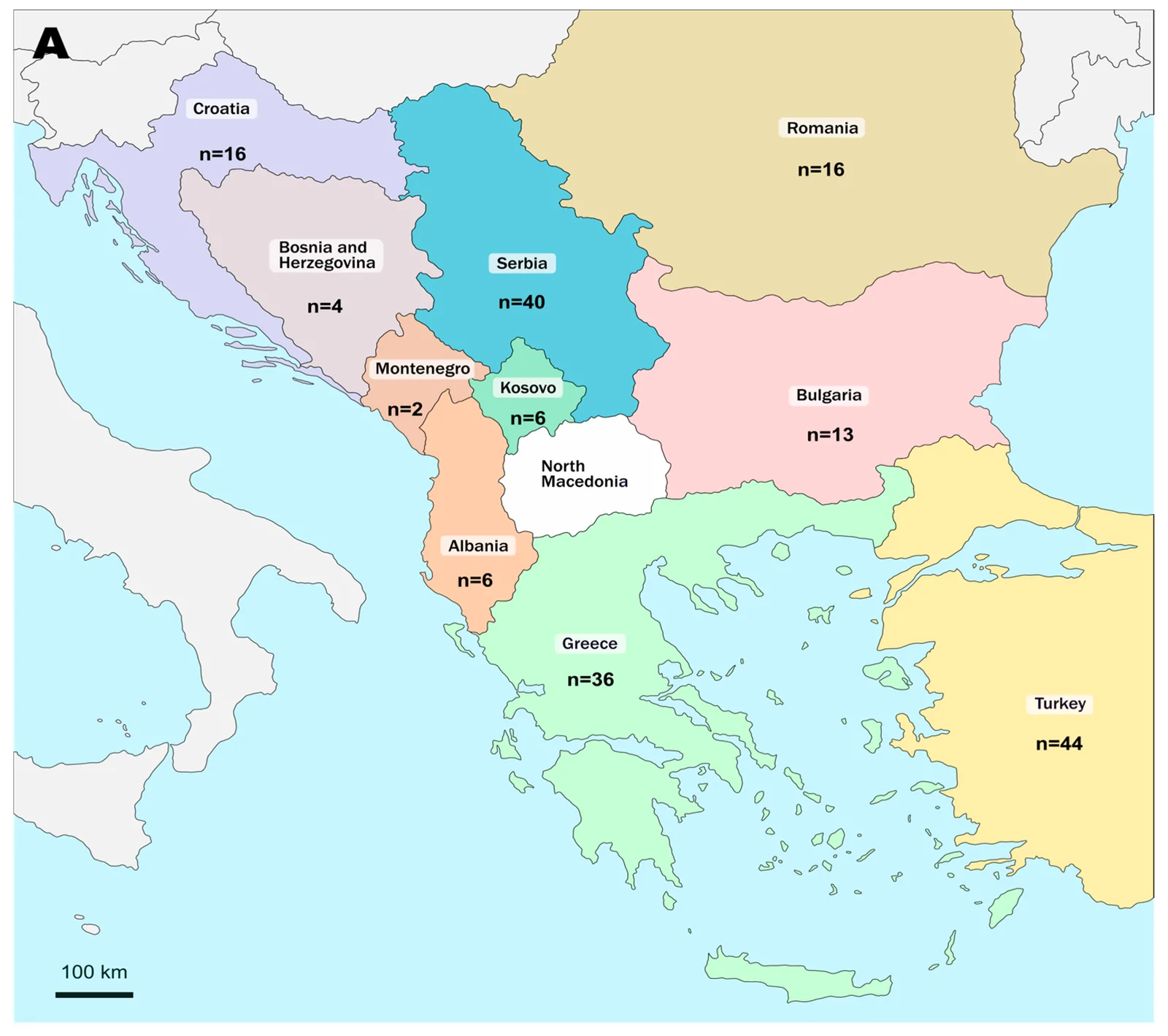

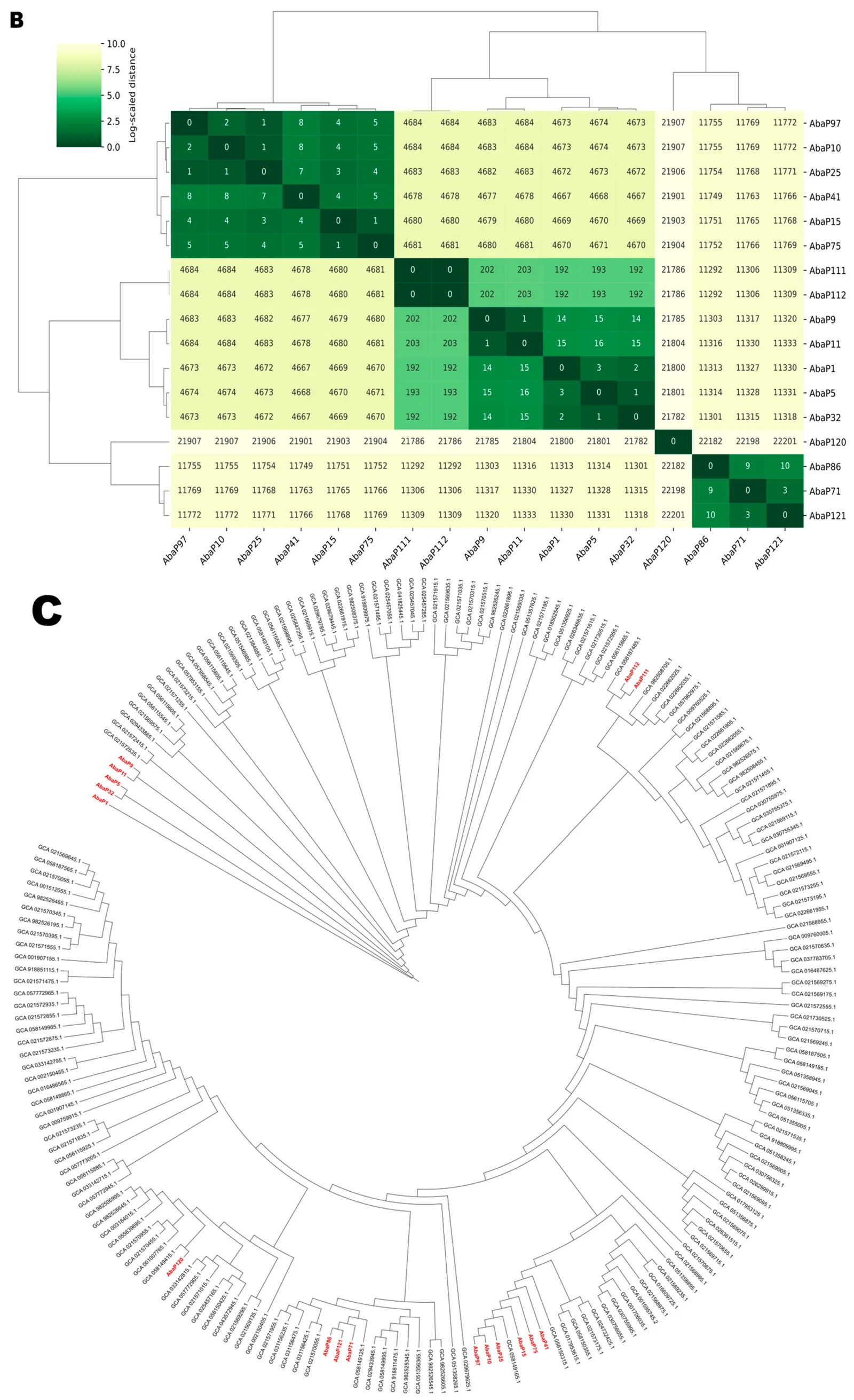
Phylogenomic analysis of clinical *A. baumannii* isolates from the Balkans retrieved from the NCBI Pathogen Detection system. (**A**) Geographic origins of the included isolates. (**B**) Hierarchical clustering based on the SNP distance matrix of the isolates analyzed in this study. (**C**) Maximum-likelihood phylogenetic tree reconstructed from single-nucleotide polymorphisms (SNPs) identified in the core genome alignment generated by pangenome analysis. Isolates sequenced in this study are highlighted in red.

To investigate the epidemiological context of the studied isolates, a collection of 200 non-duplicate clinical *A. baumannii* genomes representing isolates recovered from 11 Balkan countries was assembled as described above. The geographic distribution of these strains is shown in Figure 1A.

Hierarchical clustering of the SNP distance matrix revealed that the isolates analyzed in this study formed three distinct clusters: cluster 1 (isolates 1, 5, 9, 11, 32, 111, and 112), cluster 2 (isolates 10, 15, 25, 41, 75, and 97), and cluster 3 (isolates 71, 86, and 121) (Figure 1B). Pairwise SNP differences within these clusters ranged from 1 to 16 SNPs, indicating close relatedness, with the exception of isolates 111 and 112, which were genetically identical in this assay (0 SNP differences) but differed from the remaining members of cluster 1 by up to 203 SNPs. Isolate 120 remained the only isolate separated by more than 20,000 SNPs from all other isolates, indicating that it is not closely related to any of them.

The phylogenetic tree (Figure 1C) revealed that the closest relative of cluster 1 was genome GCA_021572415, which was recovered from a patient at Henry Dunant Hospital in Athens, Greece. The closest relatives of clusters 2 and 3 were genomes GCA_058149165.1 and GCA_058149125.1, respectively. Both genomes were generated as part of the 2026 ECDC genomic survey of carbapenem-resistant *A. baumannii* in Europe; however, no additional epidemiological information is currently available.

## 4. Discussion

### 4.1. Carbapenem Resistance Associated with blaOXA-23-like and blaOXA-72

Our data revealed the relationship between genotypic and phenotypic resistance profiles of 36 *Acinetobacter baumannii* invasive isolates recovered from blood cultures. The tested isolates showed a high prevalence of carbapenem resistance (94.4%). They were positive for *bla*_OXA-23-like_ (29/36) and *bla*_OXA-72_, a *bla*_OXA24/40-like_ variant (5/36). No isolate harbored both genes simultaneously. The *bla*_OXA-58-like_, *bla*_KPC-like_, and metallo-β-lactamase genes (*bla*_NDM_, *bla*_IMP_, *bla*_VIM_) were not detected.

These findings are consistent with the increasing trend of carbapenem resistance observed in Bulgaria. The first identification of OXA-23-producing isolates from our hospital was documented by Stoeva et al. [38] among isolates recovered between 1999 and 2006. However, *bla*_OXA-24/40-like_ genes were not detected during this period. Over approximately two decades, carbapenem resistance rose from 61.7% [39] to 94.4% within the same hospital setting. A similar increase from 59.4% (2004–2011) to 93–98.7% (2014–2022) was documented by Strateva et al. in a multicenter study [40]. The same authors reported a high prevalence of *bla*_OXA-24/40-like_ as the sole acquired carbapenem-hydrolyzing class D β-lactamase (CHDL) in isolates from Varna and two Sofia university hospitals during 2014–2022, indicating expansion of strain lineages producing this enzyme. In addition, they identified strains co-producing both CHDLs, which were not observed in our study. Across the Balkans, various surveys report *bla*_OXA-24/40-like_ (including *bla_OXA-_*_72_) and *bla*_OXA-23-like_ occurring either as mutually exclusive determinants or in simultaneous co-occurrence [41,42,43]. These reports illustrate the dissemination of *bla*_OXA-72_ across the region.

Current ECDC surveillance data (2023) confirm a pronounced east–west gradient in carbapenem resistance among invasive *A. baumannii* isolates across Europe. Resistance rates exceed 50% throughout southern and eastern Europe (Italy 80.8%, Poland 78.2%), with striking data from the Balkans (Greece 94.6%, Romania 92.7%) compared with below 5% in northern and western Europe (Germany 3.5%, Norway 1.6%) [4].

Globally, *bla*_OXA-23-like_ remains the most prevalent acquired CHDL gene, accounting for approximately 88% of carbapenemase genes in carbapenem-resistant *A. baumannii*, while *bla*_OXA-24/40-like_ and *bla*_OXA-58_-_like_ are detected less frequently [8,35,44,45]. Unlike Greece, Turkey, and other countries in the Mediterranean region, where a temporal shift from *bla*_OXA-58_ to *bla*_OXA-23_ predominance occurred between the first and second decades of the century [45,46,47,48], Bulgaria has consistently demonstrated *bla*_OXA-23-like_ dominance throughout this period, with only sporadic detections of *bla*_OXA-58-like_ [38,40].

### 4.2. Aminoglycoside Resistance Associated with armA

In the present study, gentamicin demonstrated the highest activity among the aminoglycosides. Isolates carrying *armA* exhibited high-level resistance to all three tested aminoglycosides. In *armA*-negative, *bla*_OXA-23-like_-positive isolates, MIC values for gentamicin and tobramycin decreased substantially, whereas amikacin MICs remained elevated. In *bla*_OXA-72_-positive isolates, gentamicin and tobramycin MICs fell within the susceptibility range. Notably, *armA* was detected exclusively in combination with the *bla*_OXA-23-like_ gene. Multiple publications support the co-persistence of *bla*_OXA-23-like_ and *armA* in MDR/XDR *A. baumannii* associated with ST2 (CC2/IC2) [49,50,51,52,53]. Co-occurrence of *bla*_OXA-72_ and *armA* was not observed. Although *armA* has been reported in combination with other carbapenemases, including *bla*_OXA-72_, *bla*_NDM-1_, and *bla*_OXA-58_, these associations remain rare compared with the *armA*–*bla*_OXA-23-like_ combination [54].

APH(3′)-VIa is a highly prevalent aminoglycoside-modifying enzyme, conferring resistance to amikacin and kanamycin. In the present study, whole-genome sequencing confirmed the presence of the encoding gene, *aph(3′)-VIa*, in *armA*-negative isolates, which may explain the persistently elevated amikacin MICs despite susceptibility to gentamicin and tobramycin observed in this group. A study from Iran similarly reported a predominance of *aph(3′)-VIa* in sequence groups not carrying *armA* [55].

### 4.3. armA and Minocycline Resistance Association

A substantial revision of the CLSI breakpoints for minocycline was introduced in 2025. In the SENTRY study, the susceptibility rate of XDR *A. baumannii* isolates to minocycline was 72.7% according to the former breakpoints; re-evaluation using the updated CLSI criteria reduces this estimate to approximately 40% [56]. The impact of breakpoint revision is further illustrated in other studies [57,58].

To the best of our knowledge, this is the first study in Bulgaria reporting minocycline susceptibility data for *A. baumannii*. We observed resistance in half of our isolates, using CLSI M100, 35th edition (2025) [17] breakpoints, with a further 27.8% in the intermediate range.

Minocycline resistance in *A. baumannii* is primarily mediated by the narrow-spectrum efflux pump TetB, a member of the major facilitator superfamily. In our collection, all *tet(B)*-positive isolates exhibited high MIC values (≥8 mg/L), consistent with previous reports identifying *tet(B)* as a strong predictor of minocycline resistance [58]. Sequence analysis revealed that all *armA*-positive isolates also carried *tet(B)* and exhibited high MICs (8–24 mg/L), whereas *armA*-negative isolates uniformly lacked *tet(B)* and fell within the intermediate or susceptible range.

Applying the revised 2025 CLSI minocycline breakpoints to our isolates, the susceptibility rate fell from 50.0% to 22.2%: 10 isolates (27.8%) previously classified as susceptible were reclassified as intermediate, and one previously intermediate isolate became resistant, yielding 18 resistant (50.0%), 10 intermediate (27.8%), and 8 susceptible (22.2%). Because inappropriate initial therapy is an independent predictor of mortality in XDR *A. baumannii*, interpretation under the former breakpoints could have led to falsely reassuring susceptibility reports and inappropriate use of minocycline, with an attendant risk of treatment failure and poor outcome. Based on the aforementioned findings, *armA* may serve as a reliable surrogate marker for minocycline resistance in this hospital setting, given the clear delineation of the resistance category. Furthermore, the identification of genotypes 4 and 5, which carry the *bla*_OXA-72_ gene, may predict true susceptibility to the agent. However, this association reflects the clonal structure of the local *A. baumannii* population and may not be generalizable to other settings.

### 4.4. Determinants of Trimethoprim/Sulfamethoxazole and Fluoroquinolone Resistance

The *sul1* gene, part of a class 1 integron, confers resistance to the sulfonamide component of trimethoprim/sulfamethoxazole [59]. The MIC variability among our trimethoprim/sulfamethoxazole-resistant isolates may be explained by additional mechanisms, including active efflux by RND- and MFS-type pumps, as well as multiple copies of *sul1* [60,61,62]. The absence of all investigated resistance genes in one isolate corresponded to full susceptibility to trimethoprim/sulfamethoxazole.

Both *bla*_OXA-23-like_/*armA*-positive and *bla*_OXA-24/40-like_-positive isolates harbored dual QRDR mutations in *gyrA* and *parC*. However, the former exhibited markedly higher levofloxacin MICs. This discordance suggests the involvement of additional resistance mechanisms that may differ between the two clonal lineages, ST2 and ST636 [63].

### 4.5. Cefiderocol Susceptibility in a Subset of Nine Isolates

Five of the nine isolates tested exceeded the ECOFF (0.5 mg/L) and were therefore classified as non-wild-type, indicating reduced cefiderocol activity relative to the wild-type population. All five were *armA*-negative. Notably, only two of them (MIC 4 mg/L) exceeded the clinical resistance breakpoint (R > 2 mg/L), meaning the remaining three would be reported as susceptible despite already showing a non-wild-type phenotype, an early signal of emerging resistance that routine susceptibility reporting would not capture. Given the limited size of the subset, these observations remain preliminary and require confirmation in a larger series. Detailed values are presented in Table S4 (Supplementary Materials).

### 4.6. Genotypic–Phenotypic Profiles

By correlating detected resistance genes with the MIC values for each isolate, distinct genotypic–phenotypic resistance profiles were established (Table 3 and Table 5). The most prevalent genotypic profiles were as follows: GT1 (36.1%) > GT2 (30.6%) > GT3 (13.9%) > GT4 (11.1%), reflecting the composition of the diversity-selected cohort rather than natural clinical prevalence. Overall, *bla*_OXA-23-like_ was present in 80.6% of isolates, the combination of *bla*_OXA-23-like_ with *armA* in 50.0%, and *bla*_OXA-23-like_ with *sul1* in 66.7%.

### 4.7. Current Treatment Options for Multidrug-Resistant A. baumannii Infections

The present study comprised 34/36 XDR *A. baumannii* isolates. XDR *A. baumannii* is recognized as a WHO critical-priority pathogen, associated with high treatment failure rates and mortality ranging from 18% to 89%, depending on the infection source [64]. For this reason, it is worth noting that treatment options for infections caused by XDR strains remain limited. A few agents, mostly used in combination, have been proposed. Sulbactam–durlobactam combined with a carbapenem is now recommended as first-line therapy, whereas high-dose ampicillin–sulbactam represents an alternative combined with a second active agent such as polymyxin B, minocycline, tigecycline, or cefiderocol [65,66]. Colistin, although retaining full in vitro activity in our cohort, should likewise be used in combination rather than as a monotherapy, given the risk of resistance emergence and treatment failure [48]. The revised, more conservative CLSI (2025) [17] minocycline breakpoints support its use with optimized, PK/PD-guided dosing in combination regimens, thereby reducing the risk of treatment failure [67,68]. Given the severity of XDR *A. baumannii* infections and the need for individualized treatment regimens, the genotype–phenotype associations proposed in this study, combining *bla*_OXA-23-like_, *bla*_OXA-24/40-like_, and *armA* as markers for resistance to aminoglycosides and minocycline, may contribute to a better understanding of local epidemiology and support treatment decision-making within a hospital setting.

### 4.8. Practical Recommendations

Based on our findings, the following practical recommendations can be proposed. In settings with a high prevalence of *bla*_OXA-23-like_/*armA* co-carriage, empirical avoidance of minocycline may be warranted. Given colistin susceptibility in this cohort, colistin-based combination regimens remain a viable empirical option for XDR *A. baumannii* in this hospital setting. Combined PCR screening for *bla*_OXA-23-like_, *bla*_OXA-24/40-like_, and *armA* could serve as a practical tool to guide antibiotic selection in laboratories lacking full genomic sequence capacity.

### 4.9. Phylogenetic Analysis

Phylogenetic analysis identified three clusters, corresponding entirely to the proposed genotypes. Clusters 1 and 2 comprised genotypes 1–3. All belonged to ST2 and were *bla*_OXA-23-like_-positive, distinguished by *armA* presence, highlighting the value of *armA* as a sensitive marker for specific resistance phenotypes. The single genotype 6 isolate also clustered here as an ST2 member but was *bla*_OXA-23-like_-negative and carbapenem-susceptible (discussed below). Cluster 3, positive for *bla*_OXA-72_ and *armA*-negative, belonged to ST636, a triple-locus variant of ST2.

ST2 (CC2/IC2) is the dominant *A. baumannii* lineage worldwide [69,70], strongly linked to carbapenem resistance via *bla*_OXA-23-like_ [69], and typically shows high multidrug/extensive drug resistance beyond carbapenems [49]. Notably, an Italian study of ST2 isolates causing bloodstream infections in COVID-19 patients found uniform resistance not only to carbapenems but also to colistin across all 43 strains [71]. In addition, all but one of the tested ST2 isolates from Ukrainian combat wounds were reported to be cefiderocol-resistant [72]. These two investigations highlight the potential of the clone to acquire a pan-resistant phenotype.

ST636 is mainly reported in Southeast Europe, ranking third among European STs after ST2 and ST1, and has not been reported outside Europe [73]. Furthermore, the clone is uniformly associated with *bla*_OXA-72_ production [37], consistent with our finding. In addition, plasmid localization of *bla*_OXA-72_ within ST636 has been reported [35,49]. In our collection, *bla*_OXA-72_ was confined to ST636 isolates lacking *bla*_OXA-23-like_, whereas *bla*_OXA-23-like_ was restricted to ST2, with no co-occurrence of the two determinants. This contrasts with reports of *bla*_OXA-23-like_/*bla*_OXA-72_ co-occurrence within a single clone, described both in ST2 in neighboring Romania [41] and in an ST636 isolate from Varna, Bulgaria [49]. Our ST636/*bla*_OXA-72_ profile more closely resembles the OXA-72-producing ST636 lineage reported in Serbia, both in a neonatal ICU outbreak [74] and among nationally circulating isolates [75]. However, the isolates from the Serbian studies were not subjected to WGS and therefore could not be included in our phylogenomic comparison. This suggests that the ST636/*bla*_OXA-72_ combination has arisen independently in more than one genetic background across the region, rather than spreading from a single common ancestor. ST2 and ST636 co-circulate across the Balkans, sometimes within the same hospital [41,49,75], confirmed by the results of our study.

The close phylogenetic relation of our cluster 1 isolates to a reference genome from Athens, Greece, suggests regional spread of the resistant ST2 clone across the Balkans. Isolates 111 and 112, genetically identical, were collected from the abdominal surgery department (November 2024) and ICU1 (December 2024), respectively, suggesting strain transfer between these territorially close, patient-exchanging departments.

One of the two carbapenem-susceptible isolates, 15 (cluster 2, genotype 6), may be a variant or defective strain within the cluster, while the other, 120 (genotype 7), belongs to no cluster. The significance of such strains remains unclear within this study and requires investigation of a greater number of carbapenem-susceptible isolates.

### 4.10. Limitations of the Study

This study has several limitations. First, the small number of isolates, 36 with only 17 sequenced, limits the statistical power of the gene–phenotype associations. Second, isolate selection prioritized phenotypic diversity over random sampling, so the reported genotype frequencies (e.g., GT1–GT4) reflect this diversity-enriched subset rather than true prevalence among bloodstream *A. baumannii* isolates. Third, this was a single-center, retrospective study. Consequently, certain information regarding the clinical course of the disease and the precise duration of antibiotic exposure prior to isolate collection could not be reliably reconstructed from medical records. In addition, only bloodstream isolates were included, which may limit generalizability, although bloodstream infections represent the most life-threatening manifestation of *A. baumannii* resistance. Finally, the limited number of isolates tested for cefiderocol could not provide conclusive information regarding the susceptibility of isolates in our hospital setting to the agent. Larger, multicenter studies are needed to confirm and extend these findings.

## 5. Conclusions

At present, whole-genome sequencing remains predominantly a research tool, with limited implementation in routine clinical microbiology. Routine laboratory practice requires practical and accessible approaches that can inform individualized treatment decisions and facilitate the identification of clonal lineages during outbreak investigations and surveillance of endemic strains. The seven genotypic–phenotypic profiles proposed in this study, together with the role of *armA* as a potential marker for resistance phenotypes, including not only gentamicin and tobramycin but also minocycline, represent a new contribution to the existing data on *A. baumannii* clinical isolates in Bulgaria. Combined with susceptibility testing, they may serve as a practical tool for both clinical decision-making and local epidemiological practice. It is worth mentioning that cefiderocol, as one of the few treatment options for XDR *A. baumannii* infection, requires thorough investigations with clinical isolates. Future investigations addressing pathogenic potential, such as virulence factors, biofilm formation, and closer correlation with clinical data, may further expand the knowledge and practical application of these findings.

## Figures and Tables

**Table 1:** PCR primer sequences and expected amplicon sizes used for antimicrobial resistance gene detection in this study.

Target	Primer Sequence (5′–3′)	Product Size (bp)	Reference
*bla*_OXA-23-like_	F: GATCGGATTGGAGAACCAGA R: ATTTCTGACCGCATTTCCAT	501	[19]
*bla*_OXA-24/40-like_	F: GGTTAGTTGGCCCCCTTAAA R: AGTTGAGCGAAAAGGGGATT	246	[19]
*bla*_OXA-58-like_	F: AAGTATTGGGGCTTGTGCTG R: CCCCTCTGCGCTCTACATAC	599	[19]
*bla*_NDM-like_	F: AACACAGCCTGACTTTCG R: TGATATTGTCACTGGTGTGG	111	[20]
*bla*_VIM-like_	F: GATGGTGTTTGGTCGCATA R: CGAATGCGCAGCACCAG	390	[21]
*bla*_IMP-like_	F: TTGACACTCCATTTACTGCTA R: TCATTTGTTAATTCAGATGCATA	172	[19]
*bla*_KPC-like_	F: TCTGGACCGCTGGGAGCTGG R: TGCCCGTTGACGCCCAATCC	399	[22]
*armA*	F: ATTCTGCCTATCCTAATTGG R: ACCTATACTTTATCGTCGTC	315	[23]
*sul1*	F: ACGGTGTTCGGCATTCT R: TTTGAAGGTTCGACAGC	580	[24]
IS*Aba1-bla*_OXA-23-like_	F: CACGAATGCAGAAGTTG R: ATTTCTGACCGCATTTCCAT	1404	This study

F, forward primer; R, reverse primer; bp, base pairs; IS, insertion sequence. Gene abbreviations are as defined in the main text.

**Table 2:** Antimicrobial susceptibility of the 36 *A. baumannii* isolates, n (%).

Category	MEM	IPM	GEN	AMK	TOB	LEV	SXT	MIN	COL
Susceptible	2 (5.6)	2 (5.6)	16 (44.4)	2 (5.6)	6 (16.7)	1 (2.8)	1 (2.8)	8 (22.2)	36 (100)
Intermediate	–	–	–	–	–	13 (36.1)	–	10 (27.8)	–
Resistant	34 (94.4)	34 (94.4)	20 (55.6)	34 (94.4)	30 (83.3)	22 (61.1)	35 (97.2)	18 (50.0)	0 (0)

n, number of isolates; MEM, meropenem; IPM, imipenem; GEN, gentamicin; AMK, amikacin; TOB, tobramycin; LEV, levofloxacin; SXT, trimethoprim/sulfamethoxazole; MIN, minocycline; COL, colistin. Dashes (–) indicate that no intermediate category is defined for that agent by the applied breakpoint standard. Susceptibility was interpreted according to EUCAST v16.0 clinical breakpoints, except for minocycline (CLSI M100, 35th ed., 2025) [17].

**Table 3:** Relationship between genotypic and phenotypic resistance profiles in the 36 *A. baumannii* isolates (MIC in mg/L).

Genotype	Genotypic Resistance Profiles	Phenotypic Resistance Profiles (MIC in mg/L)								No.	Percent
		MEM	IPM	GEN	AMK	TOB	LEV	SXT	MIN		
GT1	*bla*_OXA-*23*_+ bla_OXA-*24*_− armA+ *sul1*+	*≥32*	≥32	≥1024	≥256	≥1024	1–32	≥32	12–24	13	36.1%
GT2	*bla*_OXA-*23*_+ bla_OXA-*24*_− armA− *sul1*+	*≥32*	≥32	2–6	96–256	12–24	1–8	≥32	2	11	30.6%
GT3	*bla*_OXA-*23*_+ bla_OXA-*24*_− armA+ *sul1*−	*≥32*	≥32	≥1024	≥256	≥1024	12–32	1.5–4	8–12	5	13.9%
GT4	*bla*_OXA-*23*_− bla_OXA-*24*_+ armA− *sul1*+	≥32	≥32	1.5–2	64–256	0.5–1	0.75–1	6–8	0.5	4	11.1%
GT5	*bla*_OXA-*23*_− bla_OXA-*24*_+ armA− *sul1*−	≥32	≥32	3	64	1.5	1	0.5	0.5	1	2.8%
GT6	*bla*_OXA-*23*_− bla_OXA-*24*_− armA− *sul1*+	2	1	4	8	16	1	≥32	2	1	2.8%
GT7	*bla*_OXA-*23*_− bla_OXA-*24*_− armA− *sul1*−	0.125	0.5	0.75	4	1	0.47	0.25	0.38	1	2.8%
										36	100%

No., number of isolates; MEM, meropenem; IPM, imipenem; GEN, gentamicin; AMK, amikacin; TOB, tobramycin; LEV, levofloxacin; SXT, trimethoprim/sulfamethoxazole; MIN, minocycline. +, gene detected; –, gene not detected. *bla*_OXA-23_ denotes *bla*_OXA-23-like_ and *bla*_OXA-24_ denotes *bla*_OXA-24/40-like_. MIC values are given as single values or observed ranges.

**Table 4:** Draft genome assembly metrics of the studied *A. baumannii* isolates.

Isolate	Genome Size (Mbp)	GC%	N50 (bp)	No. of Contigs (≥kbp)	ST	Alleles						
						cpn60	*fusA*	*gltA*	*pyrG*	*recA*	*rplB*	*rpoB*
1	4.24	40.08	288,607	97	2	2	2	2	2	2	2	2
5	4.09	39.69	288,607	72	2	2	2	2	2	2	2	2
9	4.24	40.10	341,347	77	2	2	2	2	2	2	2	2
10	4.19	39.80	480,198	58	2	2	2	2	2	2	2	2
11	4.28	40.39	340,884	73	2	2	2	2	2	2	2	2
15	4.04	39.40	461,212	51	2	2	2	2	2	2	2	2
25	4.13	39.35	461,311	60	2	2	2	2	2	2	2	2
32	4.24	40.18	292,960	82	2	2	2	2	2	2	2	2
41	4.11	39.29	630,075	54	2	2	2	2	2	2	2	2
71	4.06	39.53	323,167	53	636	2	1	2	2	2	1	1
75	4.11	39.25	461,102	55	2	2	2	2	2	2	2	2
86	4.10	39.80	323,167	76	636	2	1	2	2	2	1	1
97	4.42	41.25	461,411	66	2	2	2	2	2	2	2	2
111	4.00	39.50	434,910	82	2	2	2	2	2	2	2	2
112	3.93	39.01	461,078	79	2	2	2	2	2	2	2	2
120	3.93	39.67	2,183,114	15	-	3	~3	2	1	6	1	~60
121	4.10	39.89	387,851	56	636	2	1	2	2	2	1	1

ST, sequence type.

**Table 5:** Whole-genome sequencing data and genotype classification of the studied isolates.

Genotype	Isolate No.	Department	Date of Isolation	Resistance Genes	QRDR Mutations	ST
GT1	32	ICU1	November 2019	*bla*_OXA-23_, *bla*_OXA-66_, *bla*_ADC-73_, *armA*, *aph(3″)-Ib*, *aph(6)-Id*, *tet(B)*, *sul1*, *catA1*, *mphE*, *msrE*	*gyrA*: S81L; *parC*: S84L	2
	5	GEC	July 2020	*bla*_OXA-23_, *bla*_OXA-66_, *bla*_ADC-73_, *armA*, *aph(3″)-Ib*, *aph(6)-Id*, *tet(B)*, *sul1*, *catA1*, *mphE*, *msrE*	*gyrA*: S81L; *parC*: S84L	2
	111	AbS	November 2024	*bla*_OXA-23_, *bla*_OXA-66_, *bla*_ADC-73_, *armA*, *aph(3″)-Ib*, *aph(6)-Id*, *aph(3′)-VIa*, *tet(B)*, *sul1*, *catA1*, *mphE*, *msrE*	*gyrA*: S81L; *parC*: S84L	2
	112	ICU1	December 2024	*bla*_OXA-23_, *bla*_OXA-66_, *bla*_ADC-73_, *armA*, *aph(3″)-Ib*, *aph(6)-Id*, *aph(3′)-VIa*, *tet(B)*, *sul1*, *catA1*, *mphE*, *msrE*	*gyrA*: S81L; *parC*: S84L	2
GT2	41	ICU1	February 2020	*bla*_OXA-23_, *bla*_OXA-66_, *bla*_ADC-11_, *bla*_OXA-20_, *aac(6′)-Ib7*, *aph(3′)-VIa*, *aph(3′)-Ia*, *sul1*	*gyrA*: S81L	2
	75	ICU1	April 2022	*bla*_OXA-23_, *bla*_OXA-66_, *bla*_ADC-11_, *bla*_OXA-20_, *aac(6′)-Ib7*, *aph(3′)-VIa*, *sul1*	*gyrA*: S81L	2
	10	ICU1	November 2023	*bla*_OXA-23_, *bla*_OXA-66_, *bla*_ADC-11_, *bla*_OXA-20_, *aac(6′)-Ib7*, *aph(3′)-VIa*, *sul1*	*gyrA*: S81L	2
	25	ICU1	March 2023	*bla*_OXA-23_, *bla*_OXA-66_, *bla*_ADC-11_, *bla*_OXA-20_, *aac(6′)-Ib7*, *aph(3′)-VIa*, *sul1*	*gyrA*: S81L	2
	97	DSS	February 2024	*bla*_OXA-23_, *bla*_OXA-66_, *bla*_ADC-11_, *bla*_OXA-20_, *aac(6′)-Ib7*, *aph(3′)-VIa*, *sul1*	*gyrA*: S81L	2
GT3	1	ICU1	January 2019	*bla*_OXA-23_, *bla*_OXA-66_, *bla*_ADC-73_, *armA*, *aph(3″)-Ib*, *aph(6)-Id*, *aph(3′)-VIa*, *tet(B)*, *mphE*, *msrE*	*gyrA*: S81L; *parC*: S84L	2
	9	ICU1	April 2021	*bla*_OXA-23_, *bla*_OXA-66_, *bla*_ADC-73_, *armA*, *aph(3″)-Ib*, *aph(6)-Id*, *aph(3′)-VIa*, *tet(B)*, *catA1*, *mphE*, *msrE*	*gyrA*: S81L; *parC*: S84L	2
	11	ICU1	April 2021	*bla*_OXA-23_, *bla*_OXA-66_, *bla*_ADC-73_, *armA*, *aph(3″)-Ib*, *aph(6)-Id*, *aph(3′)-VIa*, *tet(B)*, *catA1*, *mphE*, *msrE*	*gyrA*: S81L; *parC*: S84L	2
GT4	71	ICU1	July 2021	*bla*_OXA-72_, *bla*_OXA-66_, *bla*_ADC-74_, *aph(3′)-VIa*, *catA1*, *sul1*	*gyrA*: S81L; *parC*: S84L	636
	86	ICU1	December 2023	*bla*_OXA-72_, *bla*_OXA-66_, *bla*_ADC-74_, *aph(3′)-VIa*, *catA1*, *sul1*	*gyrA*: S81L; *parC*: S84L	636
GT5	121	ICU1	February 2024	*bla*_OXA-72_, *bla*_OXA-66_, *bla*_ADC-74_, *aph(3′)-VIa*	*gyrA*: S81L; *parC*: S84L	636
GT6	15	ICU1	August 2021	*bla*_OXA-66_, *bla*_ADC-11_, *bla*_OXA-20_, *aac(6′)-Ib7*, *aph(3′)-Ia*, *sul1*	*gyrA*: S81L	2
GT7	120	ICU1	July 2024	*bla*_OXA-314_, *bla*_ADC-7_	none	1061 Oxf

GT, genotype; ST, sequence type; QRDR, quinolone resistance-determining region; ICU1, Intensive Care Unit 1; GEC, Department of Gastroenterology; AbS, Department of Abdominal Surgery; DSS, Department of Suppurative Surgery.

## Data Availability

The whole-genome shotgun sequencing project of 17 isolates of *A. baumannii* is deposited in GenBank under BioProject accession number PRJNA1479364.

## Supplementary Materials

The following supporting information can be downloaded at https://www.mdpi.com/article/10.3390/microorganisms14081618/s1: Table S1: Distribution of selected vs. non-selected Acb bloodstream isolates; Table S2: Demographic and clinical characteristics of the patients, MIC values, and detected resistance genes for the 36 *A. baumannii* isolates; Table S3: Statistical Analyses of Associations Between Resistance Genotypes and Antimicrobial Susceptibility Phenotypes; Table S4: Cefiderocol susceptibility results and armA status among the nine tested isolates.
