# Supplementary material for: Genotypic and Phenotypic Resistance Profiles of Invasive Acinetobacter baumannii Isolates at Bulgarian University Hospital
Source: Microorganisms. 2026 Jul 24;14(8):1618. doi: 10.3390/microorganisms14081618 (PMC13515826; doi:10.3390/microorganisms14081618)
Supplement: Supplementary file 1 [file microorganisms-14-01618-s001.zip › Table S1.pdf]

Supplementary Table S1. Distribution of selected vs. non-selected Acb bloodstream isolates

Phenotype	Total isolates	Selected (n=36)	Not selected (n=31)	% selected
Susceptible to colistin only	39	18	21	46.2%
Susceptible to gentamicin and colistin only	14	8	6	57.1%
Susceptible to gentamicin, tobramycin, and colistin	8	5	3	62.5%
Susceptible to amikacin and colistin only	2	2	0	100.0%
Susceptible to tobramycin and colistin only	2	1	1	50.0%
Susceptible to carbapenems and colistin only	1	1	0	100.0%
Pan-susceptible	1	1	0	100.0%
**Total**	**67**	**36**	**31**	**53,7%**

Susceptibility phenotypes in this table reflect routine testing (VITEK 2/disk diffusion) performed at the time of clinical isolation and were used solely to guide isolate selection (Section 2.1). Confirmatory antimicrobial susceptibility testing performed for this study (Section 2.2, gradient diffusion/broth microdilution) is reported in Table 2 and Table 3 of the main manuscript; minor differences in individual agent susceptibility counts between the two tables reflect this methodological distinction rather than data inconsistency
