# Supplementary material for: Genotypic and Phenotypic Resistance Profiles of Invasive Acinetobacter baumannii Isolates at Bulgarian University Hospital
Source: Microorganisms. 2026 Jul 24;14(8):1618. doi: 10.3390/microorganisms14081618 (PMC13515826; doi:10.3390/microorganisms14081618)
Supplement: Supplementary file 1 [file microorganisms-14-01618-s001.zip › Table S4.pdf]

Supplementary table S4: Cefiderocol susceptibility results and *armA* status among the nine tested isolates.

**Isolate No**	**Genotype**	***armA* status**	**FDC MIC (mg/L)**
1	3	*armA*+	0.5
2	3	*armA*+	0.5
10	2	*armA*–	4
15	6	*armA*–	1
43	2	*armA*–	4
71	4	*armA*–	1
99	2	*armA*–	1
111	1	*armA*+	0.25
120	7	*armA*–	1.125

FDC - cefiderocol
