# Supplementary material for: Genotypic and Phenotypic Resistance Profiles of Invasive Acinetobacter baumannii Isolates at Bulgarian University Hospital
Source: Microorganisms. 2026 Jul 24;14(8):1618. doi: 10.3390/microorganisms14081618 (PMC13515826; doi:10.3390/microorganisms14081618)
Supplement: Supplementary file 1 [file microorganisms-14-01618-s001.zip › Table_S3.pdf]

Table S3: Statistical Analyses of Associations Between Resistance Genotypes and Antimicrobial Susceptibility Phenotypes

blaOXA-23 vs. MEM_R and IMI_R (Fisher's exact test)

Carbapenemase genes vs. carbapenem resistance

Contingency Tables

OXA 23		MEM_R R	MEM_R S	Total
(+)	Observed	29	0	29
	% within row	100.0%	0.0%	100.0%
(-)	Observed	5	2	7
	% within row	71.4%	28.6%	100.0%
Total	Observed	34	2	36
	% within row	94.4%	5.6%	100.0%

χ^2 Tests

	Value	df	p
χ^2	8.77	1	.003
Fisher's exact test			.033
N	36		

Contingency Tables

OXA 23		IMI_R R	IMI_R S	Total
(+)	Observed	29	0	29
	% within row	100.0%	0.0%	100.0%
(-)	Observed	5	2	7
	% within row	71.4%	28.6%	100.0%
Total	Observed	34	2	36

Contingency Tables

OXA 23		IMI_R R	IMI_R S	Total
	% within row	94.4%	5.6%	100.0%

χ^2 Tests

	Value	df	p
χ^2	8.77	1	.003
Fisher's exact test			.033
N	36		

Contingency Tables

OXA 24		IMI_R R	IMI_R S	Total
(+)	Observed	5	0	5
	% within row	100.0%	0.0%	100.0%
(-)	Observed	29	2	31
	% within row	93.5%	6.5%	100.0%
Total	Observed	34	2	36
	% within row	94.4%	5.6%	100.0%

χ^2 Tests

	Value	df	p
χ^2	0.342	1	.559
Fisher's exact test			1.000
N	36		

Contingency Tables

OXA 24		MEM_R R	S	Total
(+)	Observed	5	0	5
	% within row	100.0%	0.0%	100.0%
(-)	Observed	29	2	31
	% within row	93.5%	6.5%	100.0%
Total	Observed	34	2	36
	% within row	94.4%	5.6%	100.0%

χ^2 Tests

	Value	df	p
χ^2	0.342	1	.559
Fisher's exact test			1.000
N	36		

Independent Samples T-Test

OXA23 vs. MEM MIC

Independent Samples T-Test

		Statistic	p		Effect Size
MEM	Mann-Whitney U	72.5	.004	Rank biserial correlation	-0.286

Note. H_a $\mu_{(+)} \neq \mu_{(-)}$

Group Descriptives

	Group	N	Mean	Median	SD	SE
MEM	(+)	29	32.0	32.0	0.00	0.00
	(-)	7	23.2	32.0	15.1	5.71

OXA23 vs. IMI MIC

Independent Samples T-Test

		Statistic	p		Effect Size
IMI	Mann-Whitney U	90.0	.647	Rank biserial correlation	0.113

Note. H_a $\mu_{(+)} \neq \mu_{(-)}$

Group Descriptives

	Group	N	Mean	Median	SD	SE
IMI	(+)	29	49.4	48.0	16.0	2.97
	(-)	7	48.2	64.0	35.4	13.4

OXA24 vs. IMI MIC

Independent Samples T-Test

		Statistic	p		Effect Size
IMI	Mann-Whitney U	32.0	.032	Rank biserial correlation	-0.587

Note. H_a $\mu_{(+)} \neq \mu_{(-)}$

Group Descriptives

	Group	N	Mean	Median	SD	SE
IMI	(+)	5	67.2	64.0	17.5	7.84
	(-)	31	46.2	48.0	19.7	3.53

OXA24 vs. MEM MIC

Independent Samples T-Test

		Statistic	p		Effect Size
MEM	Mann-Whitney U	72.5	.604	Rank biserial correlation	-0.0645

Note. H_a $\mu_{(+)} \neq \mu_{(-)}$

Group Descriptives

	Group	N	Mean	Median	SD	SE
MEM	(+)	5	32.0	32.0	0.00	0.00
	(-)	31	30.0	32.0	7.73	1.39

Fisher's exact test –*bla*$_{oxa23\text{-}like}$ (-), *bla*$_{oxa24}$ (-) – carbapenem susceptibility

Contingency Tables

		MEM_R		
Both_Genes_Absent		R	S	Total
0	Observed	34	0	34
	% within row	100.0%	0.0%	100.0%
1	Observed	0	2	2
	% within row	0.0%	100.0%	100.0%
Total	Observed	34	2	36
	% within row	94.4%	5.6%	100.0%

χ^2 Tests

	Value	df	p
χ^2	36.0	1	<.001
Fisher's exact test			.002
N	36		

Contingency Tables

		IMI_R		
Both_Genes_Absent		R	S	Total
0	Observed	34	0	34

Contingency Tables

Both_Genes_Absent		IMI_R R	IMI_R S	Total
	% within row	100.0%	0.0%	100.0%
1	Observed	0	2	2
	% within row	0.0%	100.0%	100.0%
Total	Observed	34	2	36
	% within row	94.4%	5.6%	100.0%

χ^2 Tests

	Value	df	p
χ^2	36.0	1	<.001
Fisher's exact test			.002
N	36		

armA vs. aminoglycoside and minocycline resistance

Contingency Tables

arm A		GEN_R R	GEN_R S	Total
(+)	Observed	18	0	18
	% within row	100.0%	0.0%	100.0%
(-)	Observed	2	16	18
	% within row	11.1%	88.9%	100.0%
Total	Observed	20	16	36
	% within row	55.6%	44.4%	100.0%

χ^2 Tests

	Value	df	p
χ^2	28.8	1	<.001

χ^2 Tests

	Value	df	p
Fisher's exact test			<.001
N	36		

Contingency Tables

		AMK_R		
arm A		R	S	Total
(+)	Observed	18	0	18
	% within row	100.0%	0.0%	100.0%
(-)	Observed	16	2	18
	% within row	88.9%	11.1%	100.0%
Total	Observed	34	2	36
	% within row	94.4%	5.6%	100.0%

χ^2 Tests

	Value	df	p
χ^2	2.12	1	.146
Fisher's exact test			.486
N	36		

Contingency Tables

		TOB_R		
arm A		R	S	Total
(+)	Observed	18	0	18
	% within row	100.0%	0.0%	100.0%
(-)	Observed	12	6	18
	% within row	66.7%	33.3%	100.0%
Total	Observed	30	6	36

Contingency Tables

		TOB_R		
arm A		R	S	Total
	% within row	83.3%	16.7%	100.0%

χ^2 Tests

	Value	df	p
χ^2	7.20	1	.007
Fisher's exact test			.019
N	36		

Contingency Tables

		MIN_R		
arm A		R	S_I	Total
(+)	Observed	18	0	18
	% within row	100.0%	0.0%	100.0%
(-)	Observed	0	18	18
	% within row	0.0%	100.0%	100.0%
Total	Observed	18	18	36
	% within row	50.0%	50.0%	100.0%

χ^2 Tests

	Value	df	p
χ^2	36.0	1	<.001
Fisher's exact test			<.001
N	36		

Independent Samples T-Test

armA × GEN (Mann-Whitney U)

Independent Samples T-Test

		Statistic	p
GEN	Mann-Whitney U	0.00	<.001

Note. H_a $\mu_{(+)} \neq \mu_{(-)}$

armA × AMK (Mann-Whitney U)

Independent Samples T-Test

		Statistic	p
AMK	Mann-Whitney U	27.0	<.001

Note. H_a $\mu_{(+)} \neq \mu_{(-)}$

armA × TOB (Mann-Whitney U)

- Independent Samples T-Test

		Statistic	p
TOB	Mann-Whitney U	0.00	<.001

Note. H_a $\mu_{(+)} \neq \mu_{(-)}$

armA × MIN (Mann-Whitney U)

- Independent Samples T-Test

		Statistic	p
MIN	Mann-Whitney U	0.00	<.001

Note. H_a $\mu_{(+)} \neq \mu_{(-)}$

Aminoglycoside MIC comparison (armA-negative, blaOXA-23-like-positive isolates

To account for differing susceptibility breakpoints across agents, MIC values for gentamicin (GEN), tobramycin (TOB), and amikacin (AMK) were standardized as log2(MIC/EUCAST breakpoint) for the 11 armA-negative, blaOXA-23-like-positive isolates. Differences across the three agents were assessed using the Friedman test, with pairwise comparisons by Wilcoxon signed-rank test.

Comparison	Variable(s) tested	n	Test used	p-value
AMK vs. GEN (fold-above-breakpoint)	log2(MIC/breakpoint), paired	11	Wilcoxon signed-rank	0.001
AMK vs. TOB (fold-above-breakpoint)	log2(MIC/breakpoint), paired	11	Wilcoxon signed-rank	0.001
TOB vs. GEN (fold-above-breakpoint)	log2(MIC/breakpoint), paired	11	Wilcoxon signed-rank	0.001
GEN vs. TOB vs. AMK (omnibus)	log2(MIC/breakpoint), paired	11	Friedman test	<0.0001

Group Descriptives — log2(MIC/breakpoint)

Agent	n	Median (IQR)	Min	Max
Gentamicin (GEN)	11	0.00 (0.00–0.00)	-1.00	0.58
Tobramycin (TOB)	11	2.00 (1.79–2.00)	1.58	2.58
Amikacin (AMK)	11	4.00 (3.58–4.29)	3.58	5.00

armA vs. fluoroquinolone resistance

Independent Samples T-Test. (*bla*$_{OXA23\text{-like}}$ +/armA+(-))

Independent Samples T-Test

		Statistic	p
LEV	Mann-Whitney U	13.5	<.001

Note. H_a $\mu_{OXA23+/armA+} \neq \mu_{OXA23+/armA-}$

Contingency Tables

Contingency Tables

		LEV_R		
arm A		R	S_I	Total
(+)	Observed	17	1	18
	% within row	94.4%	5.6%	100.0%
(-)	Observed	5	13	18
	% within row	27.8%	72.2%	100.0%
Total	Observed	22	14	36
	% within row	61.1%	38.9%	100.0%

χ^2 Tests

	Value	df	p
χ^2	16.8	1	<.001
Fisher's exact test			<.001
N	36		

Bla$_{OXA23+}$/sul+(-)

Independent Samples T-Test

		Statistic	p
STX	Mann-Whitney U	24.0	<.001

Note. H_a $\mu_{OXA23+/sul1+} \neq \mu_{OXA23+/sul1-}$

Oxa23+/armA- vs. Oxa24+/armA-

Independent Samples T-Test – Gentamicin (GEN)

Independent Samples T-Test

		Statistic	p
GEN	Mann-Whitney U	2.50	.003

Note. H_a $\mu_{OXA23+/armA-} \neq \mu_{OXA24+/armA-}$

Independent Samples T-Test – Amikacin (AMK)

Independent Samples T-Test

		Statistic	p
AMK	Mann-Whitney U	27.5	1.000

Note. H_a $\mu_{OXA23+/armA-} \neq \mu_{OXA24+/armA-}$

Independent Samples T-Test – Tobramycin (TOB)

Independent Samples T-Test

		Statistic	p
TOB	Mann-Whitney U	0.00	.001

Note. H_a $\mu_{OXA23+/armA-} \neq \mu_{OXA24+/armA-}$

Independent Samples T-Test – Minocycline (MIN)

Independent Samples T-Test

		Statistic	p
MIN	Mann-Whitney U	0.00	.002

Note. H_a $\mu_{OXA23+/armA-} \neq \mu_{OXA24+/armA-}$
